# Context-led capacity building in time of crisis: fostering non-communicable diseases (NCD) research skills in the Mediterranean Middle East and North Africa 

**DOI:** 10.1080/16549716.2019.1569838

**Published:** 2019-02-05

**Authors:** Peter Phillimore, Abla M. Sibai, Anthony Rizk, Wasim Maziak, Belgin Unal, Niveen Abu Rmeileh, Habiba Ben Romdhane, Fouad M. Fouad, Yousef Khader, Kathleen Bennett, Shahaduz Zaman, Awad Mataria, Rula Ghandour, Bülent Kılıç, Nadia Ben Mansour, Ibtihal Fadhil, Martin O’Flaherty, Simon Capewell, Julia A. Critchley

**Affiliations:** a School of Geography, Politics & Sociology, Newcastle University, Newcastle, UK; b Department of Epidemiology & Population Health, American University of Beirut, Beirut, Lebanon; c Department of Epidemiology, Florida International University, USA; and Syrian Center for Tobacco Studies, Aleppo, Syria; d Department of Public Health, Dokuz Eylul University, Izmir, Turkey; e Institute of Community and Public Health, Birzeit University, Palestine; f Faculté de Medecine de Tunis, Tunis, Tunisia; g Department of Epidemiology & Population Health American University of Beirut, Lebanon; and Syrian Center for Tobacco Studies, Aleppo, Syria; h Public Health Department, Jordan University of Science and Technology, Irbid, Jordan; i School of Medicine, Trinity College, Dublin, Ireland; j Brighton and Sussex Medical School, Brighton, UK; k WHO Regional Office for the Eastern Mediterranean (EMRO), Cairo, Egypt; l National Public Health Institute, Tunis, Tunisia; m Institute of Psychology, Health & Society, University of Liverpool, Liverpool, UK; n Population Health Research Institute, St George’s, University of London, London, UK

**Keywords:** Research capacity building, non-communicable diseases, conflict, insecurity, sustainability, Mediterranean, Middle East

## Abstract

**Background**: This paper examines one EC-funded multinational project (RESCAP-MED), with a focus on research capacity building (RCB) concerning non-communicable diseases (NCDs) in the Mediterranean Middle East and North Africa. By the project’s end (2015), the entire region was engulfed in crisis.

**Objective**: Designed before this crisis developed in 2011, the primary purpose of RESCAP-MED was to foster methodological skills needed to conduct multi-disciplinary research on NCDs and their social determinants. RESCAP-MED also sought to consolidate regional networks for future collaboration, and to boost existing regional policy engagement in the region on the NCD challenge. This analysis examines the scope and sustainability of RCB conducted in a context of intensifying political turmoil.

**Methods**: RESCAP-MED linked two sets of activities. The first was a framework for training early- and mid-career researchers through discipline-based and writing workshops, plus short fellowships for sustained mentoring. The second integrated public-facing activities designed to raise the profile of the NCD burden in the region, and its implications for policymakers at national level. Key to this were two conferences to showcase regional research on NCDs, and the development of an e-learning resource (NETPH).

**Results**: Seven discipline-based workshops (with 113 participants) and 6 workshops to develop writing skills (84 participants) were held, with 18 fellowship visits. The 2 symposia in Istanbul and Beirut attracted 280 participants. Yet the developing political crisis tagged each activity with a series of logistical challenges, none of which was initially envisaged. The immediacy of the crisis inevitably deflected from policy attention to the challenges of NCDs.

**Conclusions**: This programme to strengthen research capacity for one priority area of global public health took place as a narrow window of political opportunity was closing. The key lessons concern issues of sustainability and the paramount importance of responsively shaping a context-driven RCB.

## Background

There now exists a growing literature on building and strengthening research capacity in public health in low- and middle-income countries (LMICs). This paper examines one such multinational project, funded by the European Commission (EC), with a focus on building capacity for research on non-communicable diseases (NCDs) in the Mediterranean Middle East and North Africa (hereafter, the Mediterranean region). We write as participants in this project, known as RESCAP-MED (*RESearch CAPacity for Public Health in the MEDiterranean*), reflecting critically on the scope, context, and limitations of our work, which took place between 2012 and 2015 and at a time of intensifying political turmoil. By the end of the project, the entire region was embroiled in the most profound and intractable crises of our era. We return to this extraordinary context below.

The surge in NCDs worldwide is now well documented [1,2]. The resultant pressure on health systems in LMICs – which are currently ill equipped for the task of managing this epidemic – is particularly dramatic [3,4]. The transition from health services geared to control infectious disease, and oriented to maternal and child health, towards health services intended to manage also the chronic conditions of middle and older age poses enormous challenges in LMIC countries. The Mediterranean region exemplifies this global challenge due to limited health budgets, lack of experience in tackling NCDs, or lack of political leverage to prioritise addressing NCDs nationally [5–7]. Moreover, even before the present crises in the region, it was evident that the role of the state in healthcare provision was declining, with a parallel expansion of the private sector as influential neoliberal solutions to health system weaknesses prevail [8]. Though cost-effective solutions to preventing and treating chronic diseases, such as diabetes, are increasingly becoming available [9–11], ameliorating the impacts of the rising rates of NCDs will require major investment in healthcare systems where building, and in some cases re-building, health infrastructure by national governments will be critical. Nurturing the varied research skills to investigate the many factors shaping emerging disease trends will be vital for countries as they plan and set priorities to address the pressures posed by the rising incidence of NCDs. This is why capacity building for research is widely considered an important component of national efforts to respond to NCDs.

Research capacity building (RCB) has its advocates and critics, but has become an increasingly important vehicle for funders over recent decades. Most existing literature on RCB in the health field has been written by academics reflecting on their own practice, rather than policy practitioners. Some authors have looked at individual projects [12–15]. Others have reviewed a series of capacity building efforts [16]. There have also been surveys of existing capacity in specific regions (e.g. [17] on sub-Saharan Africa [18]; on South-Eastern Europe). Within this literature, several themes have recurred, notably: questions of definition, and how to bring precision to a potentially elastic term like ‘capacity building’ [13,19]; how to integrate diverse disciplines, along with the challenges associated with methodological pluralism [20]; and critical reflection on how to bring rigour to evaluate impact and outcomes [12,16,21–23]. Finally, an emerging literature also reflects on the research ethics underpinning such collaborations, as well as the wider tensions and limitations inherent in such programmes [13,16,17,24]. This paper is one of the first with a focus exclusively on RCB for health research in the Middle East and North Africa (also [25]), and among very few addressing the issue of RCB in times of war, conflict, and protracted crisis [26].

RESCAP-MED was funded under an EC Framework Programme (FP7) call titled ‘Building Sustainable Capacity for Research for Health and its Social Determinants in Low and Middle Income Countries’. As discussed later, the linking of health to social determinants was of great significance as a funder-defined framework for our efforts. The overarching purpose of this partnership was to train early- and mid-career researchers across the participating countries to conduct multi-disciplinary research on NCDs, and to consolidate a regional network to strengthen research collaboration. Such a network was also intended to aid engagement with national policymakers about the long-term implications of the NCD epidemic. Crucially, the project was not established de novo; it used as a springboard a pre-existing research network created for another EC-funded research project (MedCHAMPS) [27,28].

Given the exceptional political context of our work, our aim in this paper is evaluative and reflective, rather than prescriptive. Our primary purpose is to take this project to consider questions of RCB in times of crisis, and pertinent issues of sustainability that emerge in such a context. The problem of how to sustain momentum beyond the funding period bedevils all efforts to build capacity through time-limited programmes. Yet this general problem of any RCB project was accentuated in the context that faced RESCAP-MED. For the urgency of more immediate crises in the region threatened to overwhelm state apparatuses, pushing NCD planning priorities further down national political and policy agendas, amidst unprecedented political upheavals and large-scale population displacement. We are bound to ask (and we certainly asked ourselves) whether this ever-more volatile political context made RCB in public health all but impossible, or the attempt all the more urgent. We try to do justice to these questions below.

### Context and challenges

There is a growing epidemiological literature on NCDs in the Middle East, North Africa, and Turkey [5,29,30] which highlights regional characteristics of this global problem, as well as the related challenge for national health systems [31,32]. Some of this evidence stems from our own research in MedCHAMPS: in particular from comparative country-level analyses of epidemiological data, which depict more heterogeneous trends [6,27] than is apparent from recent Global Burden of Disease (GBD) studies of cardiovascular risk factor trends across the Middle East and North Africa (MENA) region [33]. Literature on the challenges for healthcare, health systems, and governance is also growing [34–39], and, as with the epidemiological picture, there are national particularities to take into account. However, a recurring theme concerns the gap between health needs and policy response [5], a situation exacerbated by the lack of national health research frameworks across the region, including in Turkey. Hence the need for – and challenge of – research capacity strengthening [36,40].

As noted, RESCAP-MED grew out of preceding EC-funded research, in MedCHAMPS, which likewise had a focus on NCDs. MedCHAMPS’ analyses linked together epidemiological modelling of cardiovascular and diabetes trends, using mainly existing national data sources [6,27,41], with qualitative analyses of health policies and the structure of health service provision to manage these NCDs, based on primary data collection [37]. Along with European partners, four research teams from the Mediterranean region were involved: from Ramallah (Palestine), Aleppo (Syria), Tunis (Tunisia), and Izmir (Turkey). Three of these were located within universities, the exception being the Syrian partners. The Syrian Center for Tobacco Studies (SCTS) was highly unusual if not unique in the Syrian context at the time: an independent research unit, affiliated for official purposes to an anti-smoking campaigning non-governmental organisation (NGO) as a condition of its acceptability to government. SCTS had to tread a tightrope to avoid government criticism or worse, but had built up a track record of epidemiological studies with external partners and funding. Senior researchers at the other three regional partners all had substantial experience of epidemiological research and publication and all were engaged in efforts to build up teams of their own.

While MedCHAMPS’ epidemiological modelling was made possible through the leadership of existing senior scientists, the research also relied on developing the skills of early-career researchers. Moreover, a major component of the project entailed training in the application of qualitative research skills that have been under-utilised region-wide, in favour of quantitative methods, with the exception of the Palestinian team, where the Institute of Community & Public Health (ICPH) at Birzeit University had a policy (unusual in the region) of linking qualitative with quantitative methodologies in each research project. Equally, economic evaluation skills had also been crucial but to a large degree limited. Thus, the research taking place in MedCHAMPS included of necessity an element of capacity building through research training workshops.

MedCHAMPS therefore provided a framework on which to build in RESCAP-MED, enabling research skills to be developed outside the constraints of specific project needs. Moreover, MedCHAMPS’ Mediterranean partners believed that RCB necessitated a greater number of partners within the region, to foster the networking potential that a larger grouping would offer for research planning and policy influence. This led to the inclusion of academic partners in public health in Beirut (Lebanon) and Irbid (Jordan), each with substantial epidemiological research track records and a history of prior collaboration with some of the existing MedCHAMPS network (including through an influential collaboration led by the American University of Beirut [42]). These academic partners were, crucially, supplemented by the inclusion of a small team within the World Health Organization’s East Mediterranean Regional Office (WHO-EMRO), in Cairo, greatly facilitating scope for engagement with national policymakers.

As such, RESCAP-MED brought together academic institutions in six Mediterranean countries (Jordan, Lebanon, Palestine, Syria, Tunisia and Turkey), alongside academic partners in two EU countries (UK, Ireland) and one international body (WHO-EMRO) (Table 1).

**Table 1.** List of participating institutions in RESCAP-MED.

Country	Academic Institution
Ireland	*Department of Pharmacology & Therapeutics*, Trinity College Dublin, Dublin
Jordan	*Public Heath and Community Medicine*, Jordan University of Science and Technology, Irbid
Lebanon	*Faculty of Health Sciences*, American University of Beirut, Beirut
Palestine	*Institute of Community and Public Health (ICPH)*, Birzeit University, West Bank
Syria	*Syrian Center for Tobacco Studies*, Aleppo
Tunisia	*Cardiovascular Disease Research Laboratory (CAVEPLA)*, Faculté de Médecine de Tunis, Tunisia
Turkey	*Department of Public Health*, School of Medicine, Dokuz Eylul University, Izmir
UK	*Institute of Health & Society*, Newcastle University, Newcastle *Institute of Psychology, Health and Society*, University of Liverpool, Liverpool *Population Health Research Institute*, St George’s, University of London, London
International	*World Health Organization Eastern Mediterranean Regional Office* (WHO-EMRO), Cairo, Egypt

During the three years of this project (2012–15), each of the six Mediterranean partner countries was profoundly affected by the repercussions of the uprisings which started in 2011. This was most directly the case in Syria and Tunisia. But Jordan, Lebanon, and Turkey experienced massive consequences as the Syrian tragedy developed, especially through the overwhelming scale of the refugee crisis as Syrians fled their war-torn country. Moreover, the post-2011 governments in Tunisia also faced their own refugee crisis, created by the collapse of government in Libya in 2012. Only Palestine had a degree of insulation from these wider convulsions (and above all the refugee crisis), obliged instead to manage an ever-deteriorating situation under Israel’s long occupation.

## Methods

The design of RESCAP-MED sought to link two main kinds of activity. The first involved developing core research skills among early- and mid-career researchers. This focused on developing methodological skills associated with particular disciplines or fields, as well as the skills required for publishing papers in peer-reviewed journals. Short training workshops (of three to five days) were supplemented by a visiting fellowship programme for more sustained mentoring as the main means to fulfil these aims. The requirement to address the social determinants of health shaped our approach to the range of methodological skills to incorporate, and led us to prioritise five disciplines or fields that could be seen as essential components of a broad methodological toolkit for studying NCDs and their causes and consequences: epidemiology, health economics, environmental health, medical anthropology, and health policy evaluation. At the same time, it had become apparent at the outset that project partners themselves lacked knowledge about existing research capabilities and shortages relevant to NCDs in other institutions within their own countries. An empirical baseline assessment of each country’s training needs in NCD research was therefore identified as the initial project task.

**Figure 1.**
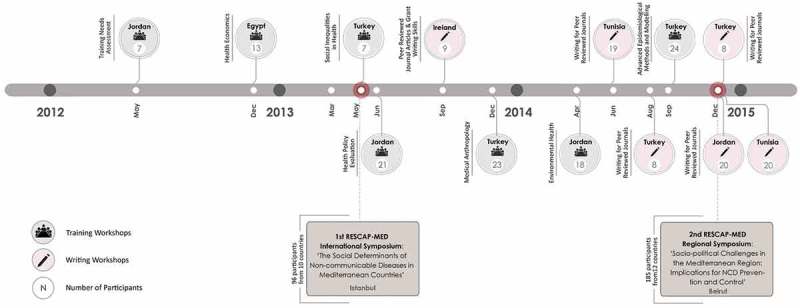
RESCAP-MED activities, locations, and number of participants.

In parallel, RESCAP-MED’s second set of activities involved the consolidation of a network with a public face for both wider academic and policy audiences in and beyond the partner countries. One aim was to assist the building of networks to foster wider research collaboration over the longer term. A second was to build the collective credentials to intensify engagement with health ministries and policymakers about research evidence and implications, policy options, and emerging research priorities. One of the chosen means to accomplish this was through holding conferences that brought together academics and policymakers from around the region, and to that end two major symposia were held in Istanbul (2013) and Beirut (2014). We also piloted an e-learning resource to disseminate teaching materials and to function as a news hub for NCD research around the region. This offered one way to sustain the momentum of capacity building beyond the funding period, and resulted in the creation of a web presence through the North Africa, Eastern Mediterranean and Turkey Public Health Network (NETPH; http://www.netph.sgul.ac.uk/). The overall project framework of activities is summarised in Figure 1.

## Results: examining achievements

While RESCAP-MED had identified the range of disciplines and fields to cover in training workshops, we devised a training needs assessment (TNA) for partners to extend their knowledge of perceived gaps and training needs within their countries. TNA were completed in Jordan, Lebanon, Palestine, Tunisia, and Turkey (see [43] for Turkish analysis and overall design of TNA). Syrian counterparts completed the first phase of the TNA, the mapping of health institutions, but were forced to discontinue the remaining phases as the political and security situation in Syria deteriorated in 2012. Training needs reported by senior and early-career researchers varied widely by country, but with consistent emphasis on enhancing quantitative skills and writing for publication. These findings led to adjustments to the workshop programme as the project unfolded, including the provision of additional epidemiological training. Moreover, as the political crisis across the region deepened, partners judged it imperative to reflect emerging health issues in the design of the later discipline-based workshops.

Despite an increasingly difficult political environment, RESCAP-MED held a total of 13 workshops. Seven of these provided discipline-based or subject-specific training (attracting 113 participants) (see Table 2, Figure 1). We planned these discipline-based workshops with two kinds of participants in mind. First, we hoped that partners would select individual staff to participate in most or all of the workshops. This regular participation would allow for incremental development of methodological skill and experience, as well as an appreciation of the complementarity of different perspectives and disciplines. Second, we hoped to attract researchers from outside partner institutions as well as government researchers to attend particular workshops. These expectations proved over-ambitious. For some partners, teams were too small to permit the same staff to attend every workshop, given day-to-day work commitments. In addition, the assumption that there might be a cadre of government-based researchers able to attend occasional workshops proved fanciful. However, while synergies across discipline-based workshops proved more limited than initially hoped, dialogue between participants within workshops was readily fostered, while the parallel activities of the network encouraged continuity. Alongside the discipline-based workshops, 6 writing workshops were also held (with 84 participants). These workshops were intended solely for staff at the six partner institutions in the region (Table 2).

**Table 2.** RESCAP-MED outputs.

Activity	Number of activities	Number of participants
**RESCAP Activities**		
Training workshops	7	113
Writing workshops	6	84
Regional and international symposia	2	281
Fellowships	18	13
**Online presence**		
NETPH website		241 users
Facebook page		335 members
Twitter		161 followers
**Publications**		
Policy briefs		3
MedCHAMPS publications realised through RESCAP-Med		>20
Publications realised through RESCAP-Med fellowships		6
Special issue of *International Journal of Public Health*		10

The intention was that each workshop would have participants from each Mediterranean partner. Yet the political situation in Syria, changes to government travel advice (for example, by the Turkish government after kidnappings of Turkish aircrew in Lebanon), and visa problems resulted in some last-minute absences as well as rearrangements to plans. We ruled out a workshop in the UK because we anticipated difficulties ensuring visas for all participants. Visa difficulties also arose elsewhere. For instance, Tunisian participants were unable to get visas in time for a training workshop in Dublin, while Palestinian participants were only able to attend a workshop in Tunisia after high-level intervention by the Palestinian Ambassador to Tunisia.

Furthermore, one lesson from the early workshops was that we had under-estimated the need for, and value of, close mentorship on scientific writing for peer-reviewed publications. The importance of such intensive writing support cannot be under-estimated. The pressure to publish (for individuals and institutions alike) is particularly challenging for early-career researchers, as much because academic writing conventions require learning as because of working in a second language. We increased the number of writing workshops in response to this need. In a number of cases, the visiting fellowship programme allowed for progress at a workshop to be consolidated with one-to-one mentoring over a longer time; indeed the completion of scientific papers became one of the most achievable goals of these fellowships. In all, 18 visits were made by 13 researchers under the visiting fellowship programme. We had anticipated that the fellowship programme would make possible both short and more extended visits, as well as a number of exchanges between Mediterranean partners themselves. In reality, however, few of the Mediterranean partners could afford the luxury of sending staff or potential trainees for periods of one month or more, while all 18 visits completed were to partners in the UK and Ireland.

Facing outwards to a wider community, NETPH became a web-based vehicle for sharing learning resources. We had initially envisaged that the extensive training materials developed during the project would have a legacy as part of NETPH. At the project’s end our hope was to transfer NETPH to a regional partner to host and develop further. However, sustaining websites requires resources, and this task has been complicated by the precarious context all Mediterranean partners have been facing.

Two major symposia were held, the first in Istanbul hosted by RESCAP-MED’s Turkish partners at DEU Izmir, the second in Beirut hosted by Lebanese partners at AUB. These events attracted 96 and 185 participants respectively (Table 2). They offered a showcase for the latest research in the region as well as drawing the attention of policymakers and government officials. If academic development was one pillar of RESCAP-MED, then engaging policymakers was another, to press the argument that research capacity was a vital investment in the people’s health. In this respect, the RESCAP-MED network, greatly enhanced by WHO-EMRO’s involvement, utilised existing well-established links with government.

However, the difficulty RESCAP-MED faced in its later stages was not in access to policymakers but in whether policy discussion could hope to have any traction in volatile circumstances. For these were exceptional times for policy engagement around long-term health challenges. Prior to 2011, ministries of health (MoHs) in the region were starting to grapple with the NCD challenge. Thereafter NCD planning – let alone NCD research capacity – predictably disappeared from view in the face of the human crisis, with population displacement overwhelming existing health infrastructure, as previously contained infectious diseases re-emerged to become a critical issue for international relief and humanitarian agencies and governments [44–46].

## Discussion

RESCAP-MED was designed prior to the political turmoil that started in January 2011 in the Mediterranean region. We could not know then that it would prove impossible to hold any training events in Syria or that academics and potential trainees in Syria would be unable to participate in this training. In addition, deterioration in the situation in both Lebanon and Tunisia due to these conflicts at certain points necessitated rearrangement of planned training events at short notice. The hopes of Tunisian partners to fund and host a Maghreb-wide event to disseminate lessons from both MedCHAMPS and RESCAP-MED to policymakers in the francophone countries in 2015 were dashed by the insecurities created after new terrorist attacks there. Fortunately Turkish and Jordanian partners stepped into the breach by hosting a larger share of events. In short, for a project built around a set of events and exchanges, the practical task of achieving these proved more daunting than ever expected. The challenges, however, were not only logistical. As we now examine, this context raised issues highly pertinent to the sustainability of and approach to RCB in conflict settings.

### Reconsidering sustainability

We are not the first to note the tension between funding of short-term capacity building initiatives and the reality that sustained commitment, of at least 10 years, is required for meaningful capacity development of an institution [16]. The difficulties in sustaining our online web presence and its training materials, through NETPH (mentioned earlier), illustrate this problem. It is easier to nurture individual capacity than it is institutional capacity, and despite an ambition for RESCAP-MED to assist both, in practice we could only realistically claim to have assisted the former (most effectively we think through the visiting fellowships). Despite this necessary caution, most of our partners and trainees are continuing on an academic career trajectory that focuses on health in their home countries and region [47]. The network can also claim continued utility, apparent in continuing exchanges and research collaborations, with new research funded from Qatar and the UK, and approaches to epidemiological modelling of diabetes prevalence that were originally used in MedCHAMPS, being developed further.

Looking from within, as participants, a vital part of the effectiveness of RESCAP-MED as both a capacity building and research network lay in the trust which was developed between partners. As emphasised, RESCAP-MED built upon the collaboration in a predecessor EC-funded project, MedCHAMPS, a connection that proved crucial in sustaining RESCAP-MED. We suggest that RCB without such a bedrock of established relationships might have less chance of success. In turn, the MedCHAMPS publication record (40 publications in peer-reviewed journals, half of these with early-career first authors) has been greatly assisted by the opportunities that became available through RESCAP-MED. This has been vital in assisting a wide range of early-career researchers to push their work through to publication in scientific journals. In short, sustainability is not always clearly defined with immediate, direct outcomes. In a longer perspective, we argue that the groundwork has been laid and models developed that should further regional efforts to strengthen NCD research capacity. It remains necessary, however, to find solutions for enhancing research infrastructure and building institutional capacities in response to the growing burden of NCDs. Yet this is a profound challenge where health systems struggle with the immensity of the current crisis, and where civil society is under sustained pressure.

## Conclusion: towards a context-led RCB

The focus of the project on the *social determinants* of NCDs pushed partners to reconsider how RESCAP-MED should respond to the public health repercussions of the political turbulence that they were living through. ‘Business as usual’ did not seem an option, while standard methodological approaches to characterising social determinants did no justice to the complexity and precariousness of the new situations developing so rapidly in these countries, and their political determinants. The fast-changing situation thus provided an impetus to revisit the very conception of social determinants of health in contexts where policy frameworks for dealing with health inequality were virtually non-existent.

Consequently, for example, training workshops on health policy evaluation, medical anthropology, and environmental health were each rethought to include sessions to examine the magnitude of the public health challenges created by forced migration and the scale of the refugee crisis. A section was added to NETPH on ‘NCDs in Displaced Populations’. The overarching theme of the second RESCAP-MED Symposium, held in Beirut in late 2014, was intended as recognition that a greatly enlarged understanding of social determinants of NCDs was required to reflect the scale of the crisis engulfing the health systems in partner countries. Syria, at that point, had endured three years of intensifying war and misery; Tunisia was still in a state of great uncertainty; and four of our partner countries (Jordan, Lebanon, Turkey, and Tunisia) were each grappling with accommodating hundreds of thousands of refugees (and in Lebanon and Turkey, over one million). In this context it would have been a travesty to have neglected the particular implications of the current crisis for NCD care and health systems. In that spirit we framed the call for the Beirut Symposium as follows:

These developments in population health are taking place against a background of political upheaval unanticipated even a few years ago, and in countries most challenged by scarcity of resources. These countries have been profoundly affected by these seismic events, with massive and growing population movements as people flee violence or its threat. The consequences for physical and mental health of extreme insecurity and hardship, and the repercussions for fragile health systems now additionally overwhelmed by the crisis, have scarcely begun to be evaluated.

One additional major benefit of the Beirut symposium was that it was also linked to a second event, the 60th anniversary conference of the Faculty of Health Sciences at the American University of Beirut, aptly called ‘Public Health in Contexts of Uncertainty’. The two events thus reinforced each other to mutual benefit and, together, reflected a more active engagement in studying conflict and health across the region, keeping in mind Palestine’s much longer track record in studying health in the context of conflict.

Against this background [48,49], RCB in RESCAP-MED became necessarily a constant adaptation to new and developing crises. The key ‘capacity’ required of the network as a whole was adaptability, for planned activities had constantly to be rethought in the light of new circumstances. The required focus on social determinants proved an incentive as well as a limitation. It opened up a perspective that was still under-recognised in some of the partner countries, more familiar with an individualistic model of health and in some cases wary of where a focus on the social might lead. But by the same token, standard depictions of social determinants inadequately reflected the profound political dynamics at play in the different contexts of the region. Thus, the network pushed towards an integration of the social and the political in our characterisation of the wider determinants of health. In light of regional and global realities, our call would be to adopt dynamic frameworks responsive to the unfolding circumstances. Also, rigidity in implementation of funder-approved frameworks may need to give way to some flexibility. RCB could never expect to be based on a pre-planned template in such circumstances, nor lead to prescribed outcomes amidst such instability. Above all, the great challenges of working in such a context should not be used to justify avoiding RCB efforts; for data-based health knowledge becomes even more crucial, and local capacity that can produce such knowledge more urgently needed.

## Paper context

This paper highlights challenges faced in research capacity building amidst profound political turmoil in the Middle East and Turkey after the revolutions of 2011. With a focus on building capacity for research on non-communicable diseases (NCDs) and their social determinants, RESCAP-MED took place at a time when NCD planning was inevitably subordinated to the immediacy of a human crisis that was overwhelming already fragile health infrastructures. The paper discusses the dilemmas posed in such circumstances.
